# A genome-wide cross-cancer meta-analysis highlights the shared genetic links of five solid cancers

**DOI:** 10.3389/fmicb.2023.1116592

**Published:** 2023-02-03

**Authors:** Hongping Guo, Wenhao Cao, Yiran Zhu, Tong Li, Boheng Hu

**Affiliations:** ^1^School of Mathematics and Statistics, Hubei Normal University, Huangshi, China; ^2^Division of Biostatistics, University of Minnesota, Minneapolis, MN, United States

**Keywords:** solid cancers, summary statistics, shared genetic loci, meta-analysis, random effect model

## Abstract

Breast, ovarian, prostate, lung, and head/neck cancers are five solid cancers with complex interrelationships. However, the shared genetic factors of the five cancers were often revealed either by the combination of individual genome-wide association study (GWAS) approach or by the fixed-effect model-based meta-analysis approach with practically impossible assumptions. Here, we presented a random-effect model-based cross-cancer meta-analysis framework for identifying the genetic variants jointly influencing the five solid cancers. A comprehensive genetic correlation analysis (genome-wide, partitioned, and local) approach was performed by using GWAS summary statistics of the five cancers, and we observed three cancer pairs with significant genetic correlation: breast–ovarian cancer (*r*_*g*_ = 0.221, *p* = 0.0003), breast–lung cancer (*r*_*g*_ = 0.234, *p* = 7.6 × 10^−6^), and lung–head/neck cancer (*r*_*g*_ = 0.652, *p* = 0.010). Furthermore, a random-effect model-based cross-trait meta-analysis was conducted for each significant cancer pair, and we found 27 shared genetic loci between breast and ovarian cancers, 18 loci between breast and lung cancers, and three loci between lung and head/neck cancers. Functional analysis indicates that the shared genes are enriched in *human T-cell leukemia virus 1 infection (HTLV-1)* and *antigen processing and presentation (APP)* pathways. Our study investigates the shared genetic links across five solid cancers and will help to reveal their potential molecular mechanisms.

## 1. Introduction

Cancer has become one of the most fatal diseases and it poses a serious threat to human life and health. There have been ~18.1 million new cancer cases and 9.6 million cancer deaths each year (Bray et al., 2018). According to the prediction of the National Cancer Institute, the number of new cancer cases per year is expected to rise to 29.5 million, and the amount of cancer-related deaths will go up to 16.4 million by 2040. The high incidence of cancer has not only brought an enormous health burden to individuals but also caused heavy economic losses to countless families. Numerous pieces of evidence indicated widespread genetic pleiotropy and shared genetic basis among different cancers (Rashkin et al., 2020). As a few representative elements of solid cancer, breast, ovarian, prostate, lung, and head/neck cancers showed substantial heritability (ranging from 9 to 57%) in previous twin and family studies (Polderman et al., 2015; Mucci et al., 2016; Yu et al., 2017). Moreover, Jiang et al. (2019) quantified the pairwise genetic correlations of six solid cancers and found significant correlations between breast and ovarian cancers, breast and lung cancers, breast and colorectal cancers, and lung and head/neck cancers. The aforementioned conclusions demonstrate indirectly that these solid cancers may share inherited genetic mechanisms, which play important roles in cancer etiology. We would like to understand the shared genetic loci influencing the five solid cancers.

Genome-wide association studies (GWASs) have identified a number of susceptibility loci associated with each of the five solid cancers, ranging from dozens to hundreds (Buniello et al., 2019), but few of them overlap in at least two of these cancers. This indicates that rare pleiotropic loci are detected by cancer-specific GWAS. Identifying the shared genetic loci between diseases can help to reveal the underlying mechanisms driving disease etiology (Guo et al., 2020). There are mainly two strategies available to identify the shared loci in the previous literature. One strategy is based on the combination of GWASs and other scan analyses. For example, Ghoussaini et al. found pleiotropic loci located at 8q24, associated with breast, prostate, and other specific cancers by using this approach (Ghoussaini et al., 2008). Another strategy is based on a cross-cancer meta-analysis. For example, Kar et al. identified seven new loci shared by at least two of the three hormone-related cancers (breast, ovarian, and prostate); Fehringer et al. (2016) detected a novel pleiotropic locus 1q22 associated with both breast and lung cancers by performing a cross-cancer genome-wide analysis of breast, ovary, prostate, lung, and colorectal cancers. However, the pleiotropic loci identified by the above studies are still not sufficient, and this may due to the fact that the cross-cancer meta-analyses in the existing studies are based on the fix-effect model. The fix-effect model meta-analysis causes the loss of statistical power because it assumes the same real effect for each genetic variant in different studies, which is practically impossible and will inevitably yield inaccurate conclusions.

Random-effect model-based cross-trait meta-analysis methods can effectively account for the heterogeneous effect of each genetic variant by adding an additional variance term, addressing the shortcomings of fix-effect model-based meta-analysis. Here, we use the summary statistics of five solid cancers (breast, ovarian, prostate, lung, and head/neck) from the largest-to-date cancer-specific GWAS consortia, which include a total of 241,479 cases and 226,810 controls. We then estimate the genetic correlation between different cancer pairs. Furthermore, we conducted a cross-cancer meta-analysis to detect shared genetic loci between the cancer pairs using the current state-of-the-art random-effect model-based approach PLEIO (Pleiotropic Locus Exploration and Interpretation using Optimal test) (Lee et al., 2021), which enables us to properly account for the correlation of traits and the heterogeneity of variants. Finally, we perform functional analyses of pleiotropic variants to uncover the underlying biological mechanisms shared across the five solid cancers.

## 2. Materials and methods

### 2.1. Data and contributing consortia

We used the most recent GWAS summary-level data from the Breast Cancer Association Consortium (BCAC) for breast cancer (122,977 cases and 105,974 controls) (Michailidou et al., 2017), the Ovarian Cancer Association Consortium (OCAC) for ovarian cancer (25,509 cases and 40,941 controls) (Phelan et al., 2017), the Prostate Cancer Association Group to Investigate Cancer Associated Alterations in the Genome (PRACTICAL) consortium for prostate cancer (79,148 cases and 61,106 controls) (Schumacher et al., 2018), the International Lung Cancer Consortium (ILCCO) for lung cancer (11,348 cases and 15,861 controls) (Wang et al., 2014), and the Oncoarray oral cavity and oropharyngeal cancer consortium for head/neck cancer (2,497 cases and 2,928 controls) (Lesseur et al., 2016).

### 2.2. Genome-wide genetic correlations

To measure genome-wide genetic correlations for each cancer pair, we used the linkage disequilibrium (LD) score regression (LDSC) method (Schizophrenia Working Group of the Psychiatric Genomics Consortium et al., 2015). We applied pre-computed LD scores derived from ~1.2 million imputed variants from European populations that did not include the HLA region in the HapMap3 reference panel. LDSC controls for population structure using GWAS summary statistics without individual-level data.

### 2.3. Partitioned genetic correlations

We evaluated the partitioned genetic correlation across the five solid cancers within functional categories by using partitioned LDSC (ReproGen Consortium et al., 2015). We chose 11 functional categories as previously recommended (Zhu et al., 2019), including the DNase I digital genomic footprinting (DGF) region, DNase I hypersensitivity sites (DHSs), fetal DHS, intron, super-enhancer, transcription factor-binding sites (TFBS), transcribed region, and the histone markers H3K9ac, H3K4me1, H3K4me3, and H3K27ac from the Roadmap Epigenomics Project (Bernstein et al., 2010). Re-computed LD scores for variants classified in each particular annotation were used for estimating the cross-cancer genetic correlation within that functional group.

### 2.4. Local genetic correlations

We estimated local genetic correlations between each pair of cancers in 1,703 pre-specified LD-independent regions using ρ-HESS (Shi et al., 2017). The goal of this method was to detect small contiguous regions of the genome in which the genetic associations of two traits are locally concordant, and to measure the local genetic correlation and *p*-values (*p*_ρ−*HESS*_) between pairs of traits at local regions. Cancer pairs were considered to have genetic correlation at the local region if *p*_ρ−*HESS*_ passed the multiple testing correction (*p*_ρ−*HESS*_ < 0.05/1703).

### 2.5. Cross-cancer meta-analysis

For the cancer pairs with significant genome-wide genetic correlation, we conducted a pairwise cross-cancer meta-analysis by using PLEIO (Lee et al., 2021). The approach is based on a random-effect model, which can not only model genetic correlations across pairs of traits but can also correct for environmental correlations. It can seamlessly test multiple traits with various types by standardizing the effect sizes. Moreover, it maps pleiotropic loci through a variance component test and calculates statistical significance through an important sampling method. It overcomes the drawback of fixed-effect model methods such as ASSET (association analysis based on subsets) (Bhattacharjee et al., 2012). We conducted the cross-cancer meta-analysis on an Intel Xeon E5-2695 computer with the CPU operating at 2.10 GHz. This wastes ~10 min for each pair of cancers.

To separate the independent loci from the significant loci (*p* < 5 × 10^−8^), we used the clumping function in PLINK software (Purcell et al., 2007). SNPs with *p* < 1 × 10^−5^, an LD statistic *r*^2^>0.05, and a distance from the peak < 1,000 kb were assigned to the clump of that peak. Moreover, we set the NCBI human genome build 37 as the reference gene list.

### 2.6. Transcriptome-wide association studies

We performed TWAS to identify gene–tissue pairs for each of the five solid cancers and used FUSION software based on the pre-computed 48 GTEx (version 7) tissue expression reference weights (Gusev et al., 2016). LD-reference data were derived from European descendants from the 1,000 Genomes Project. For each cancer, we conducted 48 TWASs, one tissue-cancer pair at a time. The false discovery rate (FDR) Benjamin–Hochberg procedure correction was used, and a result with an FDR < 0.05 was considered to be significant.

### 2.7. Replication analysis in the UK Biobank cohort

To validate our findings, we further conducted genome-wide genetic correlation analysis and cross-cancer meta-analysis of the five solid cancer GWAS datasets with the UK Biobank cohort from the IEU GWAS database project (Matthew et al., 2021): breast cancer (ID: ieu-b-4810), ovarian cancer (ID: ieu-b-4963), prostate cancer (ID: ieu-b-4809), lung cancer (ID: ieu-b-4954), and head/neck cancer (ID: ieu-b-4912). We applied the 1,000 Genomes Project variants (Phase 3) as the reference panel. The cross-cancer meta-analysis between each pair of replication datasets was implemented using the R software RE2C (Lee et al., 2017), which is another classical random-effect model-based method that tests heterogeneous effect size between individual summary statistics.

### 2.8. Pathway enrichment analysis

To gain biology insights from the shared risk genes, we performed Kyoto Encyclopedia of Genes and Genomes (KEGG) pathway analysis using the Enrichr web server (Kuleshov et al., 2016), which is a comprehensive resource for curated gene sets and a search engine that accumulates biological knowledge for further biological discoveries. The significant criterion is that the adjusted *p*-value is < 0.05.

### 2.9. Protein–protein interaction network analysis

We used STRING v10 (Szklarczyk et al., 2015) to analyze the PPI network. The basic assumption is that if two proteins are functionally associated, they may contribute to a common biological purpose. The interaction scores were derived from different sources, including experimentally determined interaction, database annotated information, and automated text mining knowledge.

**Figure 1:**
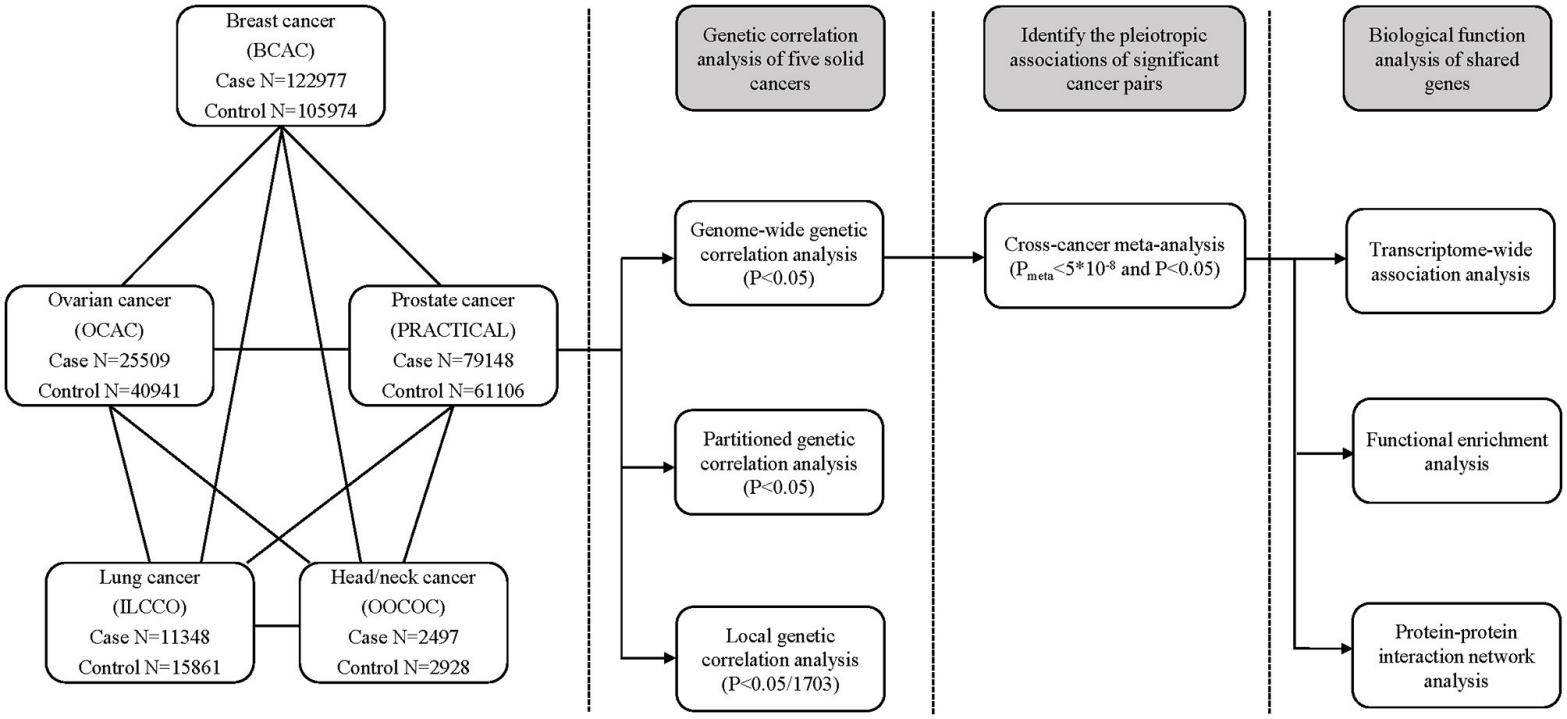
Schematic overview of the present study.

A schematic overview of the present study is shown in Figure 1, that is, we estimated genome-wide, partitioned, and local genetic correlations of the five solid cancers. For the cancer pairs with significant genome-wide genetic correlation, we performed a cross-cancer meta-analysis to identify shared genetic loci. Finally, we conducted TWAS, pathway enrichment analysis, and PPI network analysis of the shared risk genes.

## 3. Results

### 3.1. Three cancer pairs have significant genetic correlations

Among pairs of solid cancers, we found three pairs with positive genetic correlations at a significant threshold of *p* = 0.05: breast and ovarian cancers (*r*_*g*_ = 0.221, *p* = 0.0003), breast and lung cancers (*r*_*g*_ = 0.234, *p* = 7.6 × 10^−6^), and lung and head/neck cancers (*r*_*g*_ = 0.652, *p* = 0.010). The remaining pairs do not show significant genetic correlations (Table 1).

**Table 1:** Genome-wide genetic correlation between five solid cancers.

**Cancer type^a^**	**Breast**	**Ovarian**	**Prostate**	**Lung**	**Head/neck**
Breast	1	0.221	0.077	0.234	−0.065
Ovarian	0.0003	1	0.026	0.139	−0.072
Prostate	0.087	0.672	1	0.069	0.160
Lung	7.6 × 10^−6^	0.164	0.272	1	0.652
Head/neck	0.528	0.761	0.070	0.010	1

^a^The upper off-diagonal shows the genetic correlation estimates of the LD score regression (*r*_*g*_ ranges from −1 to 1), and the lower off-diagonal shows the corresponding *p*-values.

### 3.2. Most of the three cancer pairs have significant functional partitioned genetic correlations

**Figure 2:**
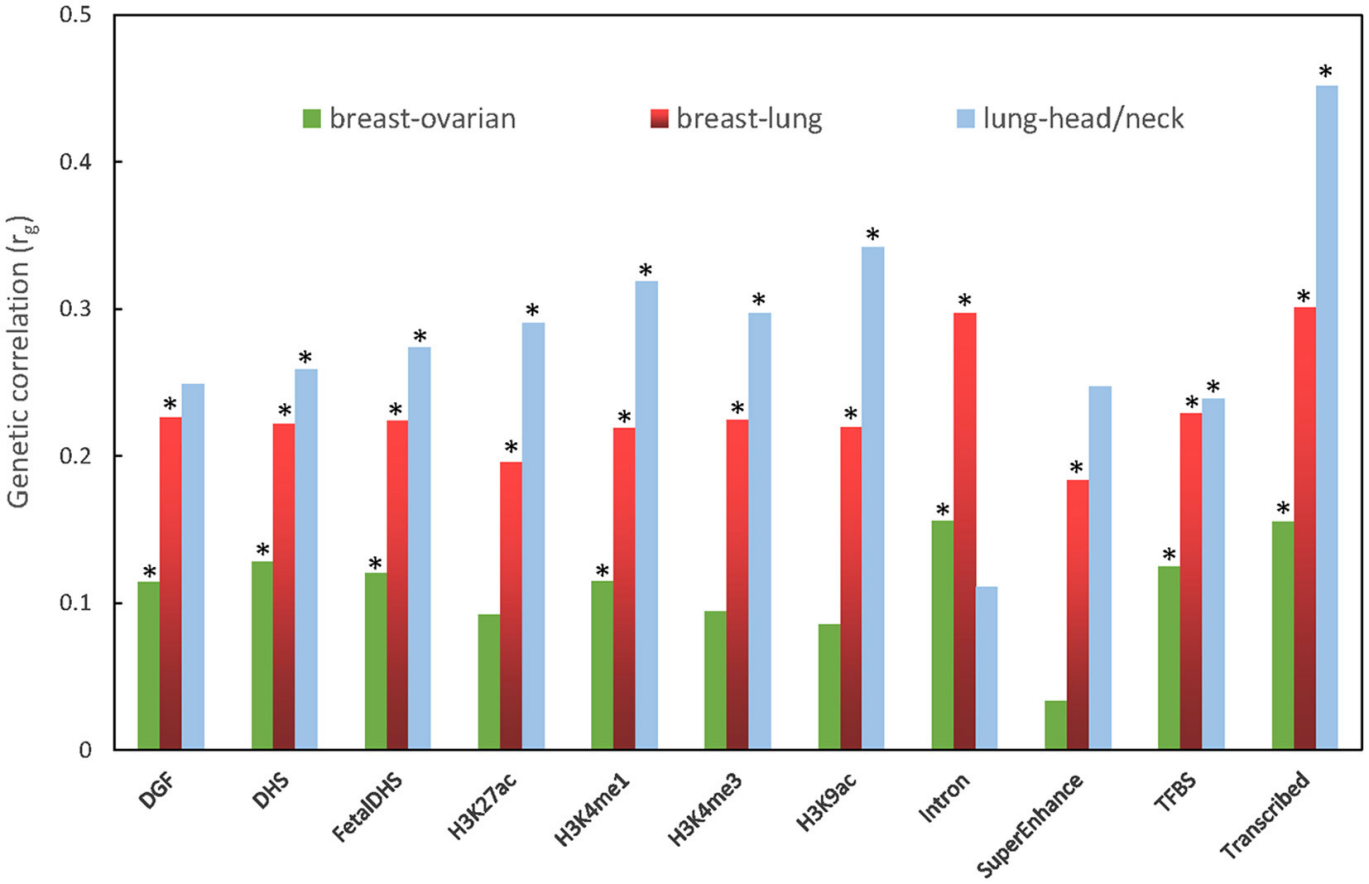
Partitioned genetic correlation between breast and ovarian cancers, breast and lung cancers, and lung and head/neck cancers. The vertical axis represents the genetic correlation *r*_*g*_, and the horizontal axis represents 11 functional categories. The asterisk represents significance (*p* < 0.05).

In the partitioned genetic correlation analysis, we observed significant genetic correlation in all 11 functional categories for the breast–lung cancer pair, with only two exceptions: Intron and SuperEnhance for the lung–head/neck cancer pair. As to the breast–ovarian cancer pair, there is no significant signal in H3K27ac, H3K4me3, H3K9ac, and SuperEnhance. The partitioned genetic correlations range from 0.033 to 0.546 (Figure 2; Supplementary Table S1).

### 3.3. Two cancer pairs have four genomic regions with significant local genetic correlations

**Figure 3:**
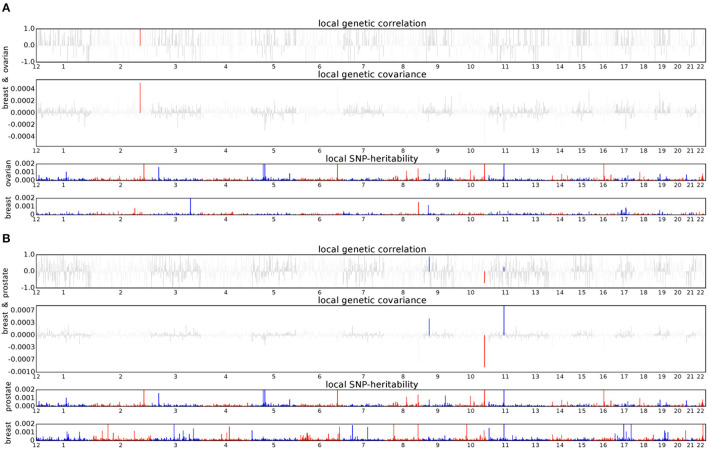
Local genetic correlation and local SNP heritability between cancer pair. **(A)** Breast and ovarian cancers; **(B)** Breast and prostate cancers. For each subfigure, the top part represents local genetic correlation, the middle part represents local genetic covariance, blue or red highlights indicate significant local genetic correlation and covariance after multiple testing corrections, and the bottom part represents local SNP heritability for each trait.

We conducted ρ-HESS to investigate whether specific regions had a genetic correlation between each pair of the five solid cancers. The results show that the breast–ovarian cancer pair has a strong local genetic correlation in the 2q33 region (chromosome 2: 201576284-202818637, *p* = 8.83 × 10^−6^) (Figure 3A). In addition, three regions, including the 9p21 region (chromosome 9: 20463534-22206559, *p* = 6.71 × 10^−6^), 10q26 region (chromosome 10: 123231465-123900545, *p* = 4.26 × 10^−7^), and 11q13 region (chromosome 11: 68005825-69516130, *p* = 4.90 × 10^−6^), are found to have strong local genetic correlations in the breast–prostate cancer pair (Figure 3B). We did not observe any significant local genetic correlations for the other cancer pairs.

### 3.4. Pleiotropic loci were identified for the three cancer pairs by cross-cancer meta-analysis

#### 3.4.1. Breast and ovarian cancer

In the cross-cancer meta-analysis, we identified 27 independent loci with a significant association between breast and ovarian cancers ($p_{meta}<5\times 10^{-8}$ and single-trait *p* < 0.05, Table 2). The strongest pleiotropic signal is mapped to *FGFR2* in the region 10q26.13 (rs1219648, $p_{meta}=4.16\times 10^{-254}$), a gene that has been altered in a number of patients with malignant solid tumors according to the AACR Project GENIE (The AACR Project GENIE Consortium et al., 2017). This SNP showed a pleiotropic association between breast and ovarian cancers according to a previous cross-cancer analysis (Kar et al., 2016). The second strongest signal is observed for chromosome 9q31.2 (rs630965, $p_{meta}=1.01\times 10^{-63}$). Patients with deletions on 9q31.2 may have delayed puberty (Iivonen et al., 2021). The third strongest signal observed on *BNC2* (rs3814113, $p_{meta}=2.16\times 10^{-43}$) is a putative tumor suppressor gene in high-grade serous ovarian carcinoma, which impacted cell survival after oxidative stress (Cesaratto et al., 2016). Notably, four loci (rs7098100, rs4277389, rs4808616, and rs10069690) are not only significant after the meta-analysis but also reach a significant level in their original single-trait GWAS.

**Table 2:** Cross-trait meta-analysis result between breast and ovarian cancers ($p_{meta}<5\times 10^{-8}$; single-trait *p* < 0.05).

**SNP**	**Genome position**	**Allele**	**Breast cancer**		**Ovarian cancer**		**Meta**	**Genes within clumping region**

			**Beta**	***p***	**Beta**	***p***	***p***	
rs1219648	chr10:123274062-123438122	A/G	0.2338	1.00 × 10^−200^	−0.0266	0.0480	4.16 × 10^−254^	*FGFR2*
rs630965	chr9:110759922-111073103	C/T	0.0992	3.21 × 10^−54^	0.0301	0.0269	1.01 × 10^−63^	CHCHD4P2^#^
rs3814113	chr9:16846323-16915021	T/C	0.0135	0.0410	−0.1780	9.40 × 10^−36^	2.16 × 10^−43^	*BNC2*
rs244353	chr17:52975892-53256579	G/A	−0.0754	1.14 × 10^−28^	−0.0295	0.0399	1.40 × 10^−31^	*COX11, STXBP4, TOM1L1*
rs6826366	chr4:175822759-175914966	G/A	−0.103	5.20 × 10^−26^	−0.0426	0.0380	2.74 × 10^−28^	*ADAM29*
rs7098100	chr10:21782842-22288132	G/A	0.0572	1.47 × 10^−18^	0.0852	6.14 × 10^−10^	4.41 × 10^−27^	*CASC10, DNAJC1, MIR1915, MLLT10, SKIDA1*
rs4277389	chr17:43513441-44865603	A/G	−0.0484	2.01 × 10^−10^	0.1151	1.20 × 10^−12^	1.20 × 10^−23^	*ARL17, CRHR1, KANSL1, LRRC37A, MAPT,MGC57346, MIR4315, NSF, PLEKHM1, SPPL2C, STH, WNT3*
rs4808616	chr19:17354825-17403033	C/A	0.0379	1.97 × 10^−8^	0.1194	8.11 × 10^−17^	1.94 × 10^−23^	*ABHD8, ANKLE1, BABAM1, NR2F6, USHBP1*
rs10069690	chr5:1279790-1279790	C/T	0.0599	7.79 × 10^−17^	0.0830	3.42 × 10^−8^	5.28 × 10^−23^	*TERT*
rs2290202	chr15:91489705-91561182	G/T	−0.0728	1.87 × 10^−15^	−0.0985	4.38 × 10^−7^	4.20 × 10^−20^	*PRC1, RCCD1, UNC45A, VPS33B*
rs851980	chr6:152008780-152070928	T/C	0.0619	1.13 × 10^−18^	0.0400	0.0083	9.44 × 10^−20^	*ESR1*
rs3769823	chr2:202119789-202271347	A/G	−0.0554	1.43 × 10^−16^	−0.0289	0.0448	1.33 × 10^−16^	*ALS2CR12, CASP8, TRAK2*
rs1474961	chr22:28324866-29318724	C/T	0.0667	1.74 × 10^−10^	−0.1091	1.80 × 10^−6^	2.02 × 10^−15^	*CCDC117, CHEK2, HSCB, MIR5739, TTC28, XBP1, ZNRF3*
rs7017073	chr8:129143680-129218127	T/C	0.0572	2.32 × 10^−14^	0.0359	0.0227	3.95 × 10^−14^	*MIR1208*
rs35958868	chr17:29164023-29247715	G/A	−0.0426	1.37 × 10^−9^	−0.0747	5.21 × 10^−7^	5.44 × 10^−13^	*ATAD5, TEFM*
rs10498635	chr14:93086918-93111120	C/T	−0.0571	3.46 × 10^−12^	−0.0748	0.0109	9.26 × 10^−13^	*RIN3*
rs381551	chr6:13638243-13722523	G/A	−0.0447	6.45 × 10^−13^	−0.0297	0.0250	2.37 × 10^−12^	*RANBP9*
rs12233670	chr4:38765720-38894380	C/T	0.0509	2.20 × 10^−12^	0.0370	0.0178	8.05 × 10^−12^	*FAM114A1, MIR574, TLR1, TLR6, TLR10*
rs2277509	chr14:91749595-91749595	C/A	0.0473	2.32 × 10^−12^	0.0296	0.0381	1.53 × 10^−11^	*CCDC88C*
rs2916074	chr19:19358672-19650096	G/A	0.0444	7.15 × 10^−12^	0.0357	0.0097	2.04 × 10^−11^	*CILP2, GATAD2A, HAPLN4, MAU2, NCAN, NDUFA13, SUGP1, TM6SF2, TSSK6, YJEFN3*
rs495828	chr9:136153875-136326248	G/T	0.0377	5.99 × 10^−7^	0.0860	9.25 × 10^−8^	7.21 × 10^−11^	*ADAMTS13, C9orf96, CACFD1, MED22, REXO4, RPL7A, SNORD24, SURF*
rs720475	chr7:144074929-144074929	G/A	−0.0488	1.20 × 10^−11^	−0.0308	0.0409	8.55 × 10^−11^	*ARHGEF5*
rs2822991	chr21:16343812-16413682	T/C	0.0533	2.44 × 10^−10^	0.0447	0.0094	5.50 × 10^−10^	*NRIP1*
rs1550623	chr2:174207470-174212894	G/A	0.0531	5.39 × 10^−10^	0.0360	0.0472	2.80 × 10^−9^	*CDCA*7^#^
rs4743687	chr9:106856452-106898410	C/T	0.0322	2.29 × 10^−7^	0.0545	4.15 × 10^−5^	4.93 × 10^−9^	*SMC2*
rs9878602	chr3:71517643-71535338	T/G	−0.0337	5.21 × 10^−8^	0.0297	0.0243	3.72 × 10^−8^	*FOXP1*
rs2941478	chr8:76474058-76476737	A/C	−0.0433	3.70 × 10^−8^	−0.0430	0.0101	4.85 × 10^−8^	*HNF4G*

^#^The nearest gene to this locus. SNP, single nucleotide polymorphisms; chr, chromosome; Allele, the character before the slash is the effect allele, and the character after the slash is the reference allele.

#### 3.4.2. Breast and lung cancers

For the breast–lung cancer pair, we detected 18 pleiotropic loci in the cross-cancer meta-analysis (Table 3). The most significant pleiotropic association is in the region 5q11.2 (rs16886181, $p_{meta}=\;4.57\times 10^{-122}$), and the mapped gene *MAP3K*1 regulates apoptosis, survival, migration, differentiation, and other functions, which suggests that it may be a target for cancer treatment (Pham et al., 2013). Moreover, we also found dense signals in the *HIST1H* gene family.

**Table 3:** Cross-trait meta-analysis result between breast and lung cancers ($p_{meta}<5\times 10^{-8}$; single-trait *p* < 0.05).

**SNP**	**Genome position**	**Allele**	**Breast cancer**		**Lung cancer**		**Meta**	**Genes within clumping region**

			**Beta**	***p***	**Beta**	***p***	***p***	
rs16886181	chr5:55983856-56306286	T/C	0.1730	8.89 × 10^−98^	−0.0670	0.0078	4.57 × 10^−122^	*MAP3K1, MIER3, SETD9*
rs2736108	chr5:1287194-1355058	C/T	−0.0622	3.88 × 10^−19^	0.0988	6.49 × 10^−5^	4.65 × 10^−24^	*CLPTM1L, MIR4457, TERT*
rs7097066	chr10:80883083-80891631	G/A	0.0765	6.18 × 10^−20^	−0.0571	0.0228	7.47 × 10^−22^	*ZMIZ1*
rs3217992	chr9:21953137-22072719	C/T	−0.0581	1.18 × 10^−19^	−0.0512	0.0227	1.78 × 10^−21^	*C9orf53, CDKN2*
rs13214023	chr6:27413924-28366151	G/A	−0.0710	1.01 × 10^−9^	0.1398	1.73 × 10^−5^	8.48 × 10^−13^	*HIST1H family, LINC01012, LOC100131289, NKAPL, OR2B, PGBD1, TOB2P1, ZKSCAN family*
rs10498635	chr14:93086918-93111120	C/T	−0.0571	3.46 × 10^−12^	0.0513	0.0292	5.24 × 10^−12^	*RIN3*
rs4971059	chr1:155148781-155666961	G/A	0.0424	4.83 × 10^−11^	0.0549	0.0041	4.36 × 10^−11^	*ASH1L, CLK2, DAP3, FAM189B, FDPS, GBA, GBAP1, HCN3, MIR92B, MIR555, MSTO1, MSTO2P, MTX1, MUC1, PKLR, POU5F1P4, RUSC1, SCAMP3, THBS3, TRIM46, YY1AP1*
rs13207082	chr6:26309908-27251379	A/T	−0.0710	2.10 × 10^−9^	0.1225	0.0002	5.13 × 10^−11^	*ABT1, BTN1A1, BTN2A, BTN3A, GUSBP2, HCG11, HIST1H, HMGN4, LINC00240, LOC285819, LOC100270746, MIR3143, PRSS16, ZNF322*
rs3117574	chr6:31081838-32064726	G/A	−0.0233	0.0286	0.1839	2.18 × 10^−10^	4.27 × 10^−10^	*ABHD16A, AIF1, APOM, ATP6V1G2, BAG6, C2, C4A, C4B, C6orf25, C6orf47, C6orf48, CCHCR1, CDSN, CFB, CLIC1, CSNK2B, CYP21A, DDAH2, DDX39B, DXO, EHMT2, GPANK1, HCG26, HCG27, HCP5, HLA-B, HLA-C, HSPA1 family, LSM2, LST1, LTA, LTB, LY6G family, MCCD1, MICA, MICB, MIR1236, MIR4646, MIR6832, MIR6891, MSH5, NCR3, NELFE, NEU1, NFKBIL1, POU5F1, PRRC2A, PSORS1C, SAPCD1, SKIV2L, SLC44A4, SNORA38, SNORD family, STK19, TCF19, TNF, TNXA, TNXB, VARS, VWA7, ZBTB12*
rs1550623	chr2:174207470-174212894	G/A	0.0531	5.39 × 10^−10^	0.0655	0.0090	7.07 × 10^−10^	*CDCA*7^#^
rs4930103	chr11:2018168-2024683	G/A	0.0382	6.60 × 10^−10^	0.0389	0.0318	1.95 × 10^−9^	*H19*
rs4635969	chr5:1308552-1308552	G/A	−0.0173	0.0276	−0.1444	5.33 × 10^−10^	2.28 × 10^−9^	*MIR*4457^#^
rs13212534	chr6:25874423-25983010	G/A	−0.0647	1.72 × 10^−7^	0.1241	0.0005	5.70 × 10^−9^	*SLC17A2, SLC17A3, TRIM38*
rs1707302	chr1:46600917-46603348	A/G	0.0364	2.95 × 10^−8^	0.0625	0.0016	7.40 × 10^−9^	*PIK*3*R*3^#^
rs13718	chr5:132384689-132444509	A/G	−0.0437	9.38 × 10^−9^	−0.0560	0.0092	9.17 × 10^−9^	*HSPA4*
rs224121	chr10:64447352-64588680	A/C	0.0396	7.38 × 10^−8^	−0.0614	0.0041	1.72 × 10^−8^	*ADO, EGR2*
rs2524005	chr6:29899677-29899677	G/A	−0.0297	0.0003	0.1080	2.12 × 10^−6^	4.17 × 10^−8^	*HLA*−*K*^#^
rs4808616	chr19:17403033-17403033	C/A	0.0379	1.97 × 10^−8^	0.0414	0.0380	4.43 × 10^−8^	*ABHD8*

^#^The nearest gene to the locus, SNP, single nucleotide polymorphisms; chr, chromosome; Allele, the character before the slash is the effect allele, and the character after the slash is the reference allele.

#### 3.4.3. Lung and head/neck cancers

A total of three loci were identified after conducting a meta-analysis of lung and head/neck cancers (Table 4). The first (rs380286, $p_{meta}=2.72\times 10^{-12}$) is mapped on *CLPTM1L* and *MIR4457*, genes encoding the catalytic subunit of human telomerase reverse transcriptase (McKay et al., 2017). The second (rs3117575, $p_{meta}=8.06\times 10^{-12}$) is in close proximity to *ABHD16A* and many other genes. *ABHD16A* is an emerging enzyme, mainly involved in lipid metabolism and intracellular signaling, leading to the metastasis of cancer (Xu et al., 2018). The third (rs2736100, $p_{meta}=\;1.09\times 10^{-9}$) is mapped on *TERT*, a gene that plays a central role in modulating telomerase activity in tumors (Colebatch et al., 2019).

**Table 4:** Cross-trait meta-analysis result between the lung and head/neck cancers ($p_{meta}<5\times 10^{-8}$; single-trait *p* < 0.05).

**SNP**	**Genome position**	**Allele**	**Lung cancer**		**Head/neck cancer**		**Meta**	**Genes within clumping region**

			**Beta**	***p***	**Beta**	***p***	***p***	
rs380286	chr5:1299213-1355058	G/A	−0.1286	3.39 × 10^−12^	0.0890	0.0332	2.72 × 10^−12^	*CLPTM1L, MIR4457*
rs3117575	chr6:31094703-32059867	T/C	0.1839	2.37 × 10^−10^	0.2990	0.0024	8.06 × 10^−12^	*ABHD16A, AIF1, APOM, ATP6V1G2, BAG6, C2, C4A, C4B, C4B_2, C6orf25, C6orf47, C6orf48, CCHCR1, CFB, CLIC1, CSNK2B, CYP21A1P, CYP21A2, DDAH2, DDX39B, DXO, EHMT2, GPANK1, HCG26, HCG27, HCP5, HLA-B, HLA-C, HSPA1A, HSPA1B, HSPA1L, LOC102060414, LSM2, LST1, LTA, LTB, LY6G5B, LY6G5C, LY6G6C, LY6G6D, LY6G6E, LY6G6F, MCCD1, MICB, MIR1236, MIR4646, MIR6832, MIR6891, MSH5, MSH5-SAPCD1, NCR3, NELFE, NEU1, NFKBIL1, POU5F1, PRRC2A, PSORS1C1, PSORS1C2, PSORS1C3, SAPCD1, SKIV2L, SLC44A4, SNORA38, SNORD48, SNORD52, SNORD84, SNORD117, STK19, TNF, TNXA, TNXB, VARS, VWA7, ZBTB12*
rs2736100	chr5:1286516-1286516	C/A	−0.1062	3.97 × 10^−9^	−0.0970	0.0210	1.09 × 10^−9^	*TERT*

SNP, single nucleotide polymorphisms; chr, chromosome; Allele, the character before the slash is the effect allele, and the character after the slash is the reference allele.

### 3.5. Overlapped gene–tissue pairs shared by cancer pairs in TWAS

To assess the association of gene expression in specific tissue between each pair of the five solid cancers, we performed TWAS. A total of 1,669 gene–tissue pairs are significantly associated with breast cancer after Benjamini–Hochberg correction (Supplementary Table S2), in addition to 418 gene–tissue pairs with ovarian cancer (Supplementary Table S3), 1,116 gene–tissue pairs with prostate cancer (Supplementary Table S4), 155 gene–tissue pairs with lung cancer (Supplementary Table S5), and 15 gene–tissue pairs with head/neck (Supplementary Table S6). Among them, 306 gene–tissue pairs are overlapped for the breast–ovarian cancer pair, and the tissues involved are scattered; however, a number of genes are almost concentrated in the clumping region of rs4277389 on chromosome 17, such as *CRHR1, LRRC37A*, and *MAPT* (Supplementary Table S7). Moreover, 23 gene–tissue pairs are overlapped for the breast–lung cancer pair, and most of the gene signals are observed in the 1q22 region, especially gene *GBAP1*, which is simultaneously significant in eight tissues (adipose, artery, breast, fibroblast cell, sigmoid colon, transverse colon, esophagus, and vagina) (Supplementary Table S7). In addition, one gene–tissue pair (*CFB-*pituitary) is overlapped for the lung–head/neck cancer pair (Supplementary Table S7).

### 3.6. Results of replication analysis in the UK Biobank cohort

In the replication analysis, we confirmed the significance of the genetic correlation between the breast and ovarian cancer pair (*r*_*g*_ = 0.175, *p* = 0.0061), the breast and lung cancer pair (*r*_*g*_ = 0.125, *p* = 0.0018), and the lung and head/neck cancer pair (*r*_*g*_ = 0.506, *p* = 0.0005) in the UK Biobank. Then, we used cross-cancer meta-analysis (RE2C) to identify the shared genes between each of the three cancer pairs. For the breast–ovarian cancer pair, nine loci showed genome-wide significance. Of these, genes *FGFR2, BNC2, ADAM29, ESR1, ATAD5*, and *TEFM* were replicated when compared with their specific consortium results (Supplementary Table S8). Moreover, six loci demonstrated significance in the breast–lung cancer pair. Some genes were found to be replicated, such as *MAP3K1* (rs12653202, $p_{meta}=4.34\times 10^{-23}$), *HIST1H family* (rs13214023, $p_{meta}=2.83\times 10^{-14}$), *ASH1L* (rs4971059, $p_{meta}=5.47\times 10^{-9}$), and *ZMIZ1* (rs7904249, $p_{meta}=1.22\times 10^{-8}$) (Supplementary Table S9). In addition, we identified two loci shared in the lung–head/neck cancer pair, but neither was replicated (Supplementary Table S10).

### 3.7. Results of biological analysis and pathway enrichment analysis

We observed shared genes enriched in *human T-cell leukemia virus 1 infection (HTLV-1)* and *antigen processing* and *presentation (APP)* pathways. *HTLV-1* was the first retrovirus discovered to cause adult T-cell leukemia (*ATL*), a highly aggressive blood cancer (Matsuoka and Jeang, 2011). The *APP* pathway is a key element for an efficient response to immune checkpoint inhibitor therapy, which can be exploited to enhance tumor immunogenicity and to increase the efficacy of immunotherapy. The use of immune checkpoint inhibitors has already shown significant clinical advances in a wide range of patients with cancer (D'Amico et al., 2022).

### 3.8. Results of protein–protein interaction network analysis

**Figure 4:**
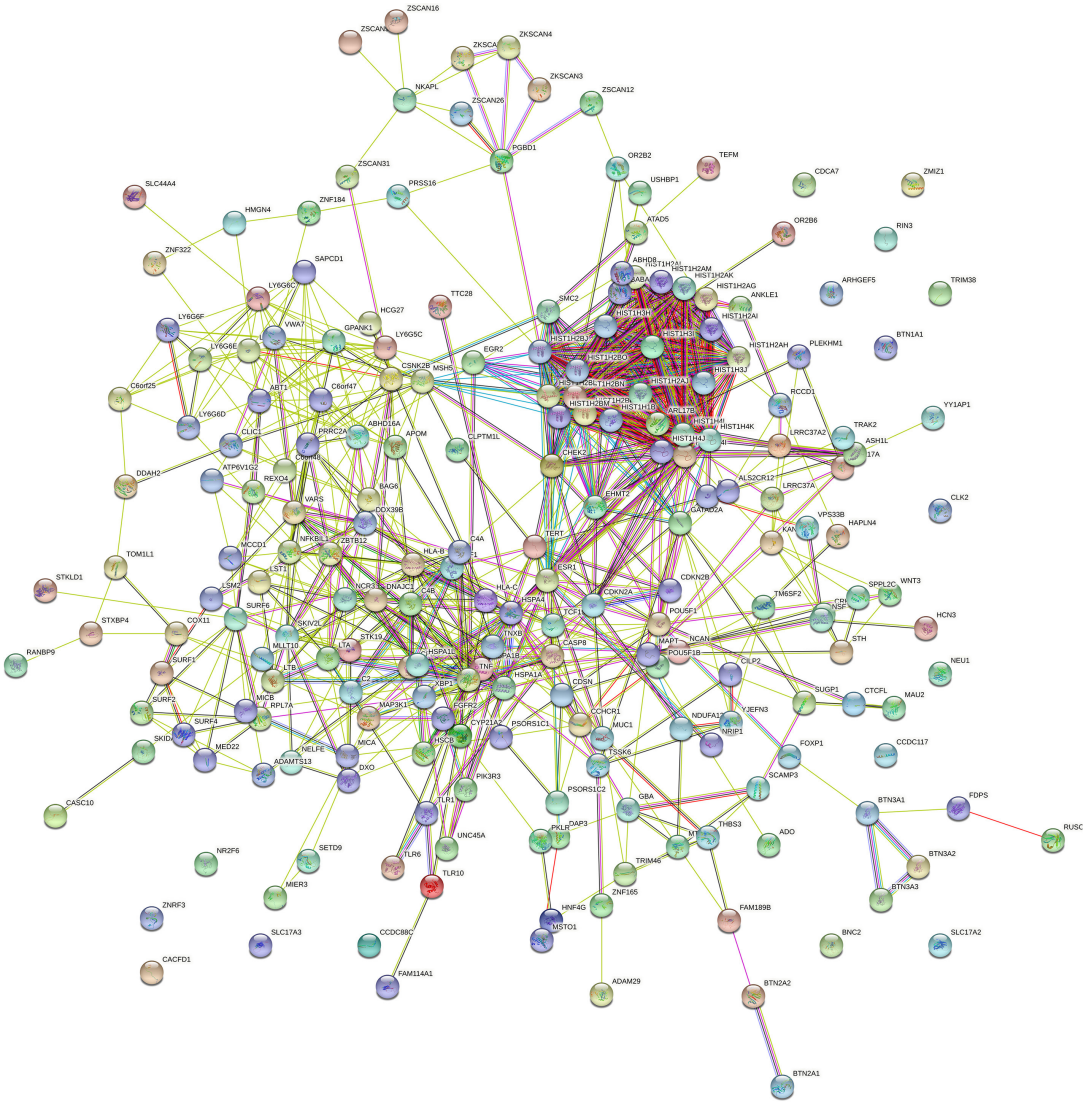
Protein–protein interaction network of share genes.

In total, we found 849 pairs of interaction in the PPI network (Supplementary Table S11). A total of 44 gene pairs have combined scores >0.95, in which the *ESR1-NRIP1* pair has the highest score of 0.999. *HIST1H* family genes around the 6p22.1 region show strong interactions with high scores. We observed 26 genes with degrees >20, most of which are *HIST1H* family genes, in addition to *ESR, HSPA4, TNF*, and *EHMT2* genes. *HIST1H* gene set expression was reported to be positively correlated with large tumor size, high grade, metastasis, and poor survival in patients with breast cancer (Liao et al., 2021), which were used as prognostic factors for survival prediction among patients with cervical cancer (Li et al., 2017). The PPI network for shared risk genes is shown in Figure 4.

## 4. Discussion

In the present study, we conducted a comprehensive analysis measuring the genetic correlation of five solid cancers, leveraging summary statistics from the current largest GWAS cancer consortia. We found significant positive genome-wide genetic correlations in three cancer pairs: breast–ovarian cancer, breast–lung cancer, and lung–head/neck cancer. Although the correlation in the prostate–head/neck cancer pair was up to 0.139, it failed to reach a significant level.

In partitioned genetic correlation, we detected positive genetic correlation and statistical significance in most function regions of the genome for the three cancer pairs, which showed significance in LDSC. Among them, the transcribed region had the strongest magnitude and significance. Most of the susceptibility variants detected by GWAS are located in non-coding regions and affect most cancers by affecting gene expression (Sud et al., 2017). Histone markers, including H3K27ac, H3K4me1, H3K4me1, and H3K9ac, are important modifications that are associated with the dysregulation of many genes that play important roles in cancer development and progression (Kurdistani, 2007). Transcribed regions have diverse transcripts that impact cancer initiation and progression through several mechanisms of action (Gibert et al., 2022).

In the analysis of local genetic correlation, we identified a novel pleiotropic region (11q13) that showed a significant local genetic correlation between breast and prostate cancers. Although the 2q33 region was previously reported as a shared region for breast–ovarian and breast–prostate cancers (Jiang et al., 2019), we only observed the pleiotropic signal in the breast–ovarian cancer pair. In addition, the 9p21 and 10q26 regions we identified were indicated to share breast and prostate cancers (Jiang et al., 2019). However, we did not find any significant local correlation between the breast–lung cancer pair and the lung–head/neck cancer pair, which showed genome-wide statistical significance.

There are some common findings in the aforementioned three kinds of genetic correlation analyses. The three cancer pairs (breast–ovarian, breast–lung, and lung–head/neck), which were significant in genome-wide genetic association analysis, also showed strong significance in most functional categories in the partitioned genetic correlation analysis (Figure 2). In addition, the breast–ovarian cancer pair also showed strong significance in the 2q33 region in the local genetic correlation analysis (Figure 3A).

In the cross-cancer meta-analysis, we discovered 27 shared loci between breast and ovarian cancers, 18 shared loci between breast and lung cancers, and three shared loci between lung and head/neck cancers. Except for four of the shared loci that showed a significant association in trait-specific GWAS of two cancers, the others were newly discovered. In contrast, a previous study, which used the fixed-effect model-based approach ASSET, only identified one novel pleiotropic association at 1q22 involved in breast and lung cancers (Kar et al., 2016). This comparison demonstrated the high statistical power of the cross-cancer meta-analysis *via* the PLEIO test, which is based on a random-effect model.

In the TWAS analysis, we explored the significant gene–tissue pair in the five solid cancers by integrating GWAS summary statistics and GTEx tissue expression data. We identified 1,669 gene–tissue pairs associated with breast cancer at the transcriptome-wide level, in addition to 418 with ovarian cancer, 1,116 with prostate cancer, 155 with lung cancer, and 15 with head/neck cancer. Furthermore, we noticed that 306 gene–tissue pairs overlapped in the breast–ovarian cancer pair, 23 pairs overlapped in the breast–lung cancer pair, and one pair overlapped in the lung–head/neck cancer pair. These overlaps may implicate specific common regulations for biological function.

In the replication analysis, we found some shared genes in two independent cohorts, such as *FGFR2* for the breast–ovarian cancer pair and *MAP3K1* for the breast–lung cancer pair. Since there are more cases (tens of thousands) in specialized cohorts (such as BCAC for breast cancer) than those in the UK Biobank cohort (nearly 1,000), the small number of cases could affect the genetic correlation estimation; this may be the reason only a fraction of pleiotropic genes were found in UK Biobank replications.

The post-GWAS analyses enabled us to provide biological insights into the shared genes. We found that the shared genes were enriched in *HTLV-1* and *APP* pathways *via* pathway enrichment analysis. In the PPI network analysis, we observed obvious aggregations around HIST1H family genes, which were proved to be used as prognostic factors for survival prediction among patients with cancer (Li et al., 2017).

There are some advantages of the present study. On the one hand, we conducted a cross-cancer meta-analysis using two large-scale cohorts for each cancer separately, which facilitated the detection of novel associations. On the other hand, we performed association analyses under two kinds of mainstream random-effect model-based methods, which confirmed some of the discoveries. We also point out the limitations of this study. First, the UK Biobank cohort cancers we used in our replication analysis are not independent because there may be some shared cases and substantial shared controls among these five solid cancers. Moreover, the identified pleiotropic loci can be divided into causal and non-causal, and further experiments are required to distinguish the causal loci and to study their biological function. Finally, our study focuses on identifying shared genetic factors across five solid cancers, and their shared environmental factors require further investigation.

## 5. Conclusion

Identifying the shared genetic loci across five solid cancers plays an important role in the etiology and pathogenesis of each cancer. Our study finds several significant genetic correlations in specific cancer pairs, and their corresponding pleiotropic variants are detected by a cross-cancer meta-analysis. We observe shared genes enriched in the *human T-cell leukemia virus 1 infection (HTLV-1)* and *antigen processing and presentation (APP)* pathways. These shared genes and pathways may help to provide clues for future drug development.

## Data availability statement

The original contributions presented in the study are included in the article/Supplementary material, further inquiries can be directed to the corresponding author.

## Author contributions

HG: conceptualization, methodology, and software. HG, WC, TL, and YZ: writing the original draft preparation. HG, WC, YZ, and BH: writing, reviewing, and editing. All authors have read and agreed to the published version of the manuscript. All authors contributed to the article and approved the submitted version.
